# S100A8/S100A9 deficiency increases neutrophil activation and protective immune responses against invading infective L3 larvae of the filarial nematode *Litomosoides sigmodontis*

**DOI:** 10.1371/journal.pntd.0008119

**Published:** 2020-02-27

**Authors:** Stefan J. Frohberger, Frederic Fercoq, Anna-Lena Neumann, Jayagopi Surendar, Wiebke Stamminger, Alexandra Ehrens, Indulekha Karunakaran, Estelle Remion, Thomas Vogl, Achim Hoerauf, Coralie Martin, Marc P. Hübner

**Affiliations:** 1 Institute for Medical Microbiology, Immunology and Parasitology, University Hospital Bonn, Bonn, Germany; 2 Unité Molécules de Communication et Adaptation des Microorganismes (MCAM, UMR 7245), Muséum national d’Histoire naturelle, CNRS; Paris, France; 3 Institute of Immunology, University Hospital of Münster, Münster, Germany; 4 German Center for Infection Research (DZIF), partner site Bonn-Cologne, Bonn, Germany; National Institutes of Allergy and Infectious Diseases, NIH, UNITED STATES

## Abstract

Neutrophils are essentially involved in protective immune responses against invading infective larvae of filarial nematodes. The present study investigated the impact of S100A8/S100A9 on protective immune responses against the rodent filarial nematode *Litomosoides sigmodontis*. S100A9 forms with S100A8 the heterodimer calprotectin, which is expressed by circulating neutrophils and monocytes and mitigates or amplifies tissue damage as well as inflammation depending on the immune environment. Mice deficient for S100A8/A9 had a significantly reduced worm burden in comparison to wildtype (WT) animals 12 days after infection (dpi) with infective L3 larvae, either by the vector or subcutaneous inoculation, the latter suggesting that circumventing natural immune responses within the epidermis and dermis do not alter the phenotype. Nevertheless, upon intradermal injection of L3 larvae, increased total numbers of neutrophils, eosinophils and macrophages were observed within the skin of S100A8/A9^-/-^ mice. Furthermore, upon infection the bronchoalveolar and thoracic cavity lavage of S100A8/A9^-/-^ mice showed increased concentrations of CXCL-1, CXCL-2, CXCL-5, as well as elastase in comparison to the WT controls. Neutrophils from S100A8/A9^-/-^ mice exhibited an increased *in vitro* activation and reduced L3 larval motility more effectively *in vitro* compared to WT neutrophils. The depletion of neutrophils from S100A8/A9^-/-^ mice prior to *L*. *sigmodontis* infection until 5dpi abrogated the protective effect and led to an increased worm burden, indicating that neutrophils mediate enhanced protective immune responses against invading L3 larvae in S100A8/A9^-/-^ mice. Interestingly, complete circumvention of protective immune responses in the skin and the lymphatics by intravenous injection of L3 larvae reversed the phenotype and resulted in an increased worm burden in S100A8/A9^-/-^ mice. In summary, our results reveal that lack of S100A8/S100A9 triggers L3-induced inflammatory responses, increasing chemokine levels, granulocyte recruitment as well as neutrophil activation and therefore impairs larval migration and susceptibility for filarial infection.

## Author summary

Human pathogenic filariae are responsible for several devastating diseases such as lymphatic filariasis and onchocerciasis. Neutrophils are essentially involved in protective immune responses against invading infective L3 larvae of filarial nematodes. The S100A8/S100A9 heterodimer, also known as calprotectin, is abundantly expressed by neutrophils and markedly increased during infection, tissue damage, neutrophil extracellular trap formation (NETosis) and cellular necrosis. However, calprotectin is also known to have an anti-inflammatory or protective role during infection and inflammation and there is an ongoing controversy on whether S100A8/A9 is pathogenic or rather protective. The present study investigated the impact of calprotectin on protective immune responses against the rodent filarial nematode *Litomosoides sigmodontis*. Investigations in *L*. *sigmodontis*-infected mice deficient in S100A8/S100A9 and wild-type controls demonstrate that the lack of S100A8/S100A9 promotes protective responses that trigger L3-induced inflammation by increasing chemokine production, granulocyte recruitment and neutrophil activation, triggering transient inflammatory responses and therefore impairing larval migration and worm recovery.

## Introduction

Inflammation represents an essential process in the initiation, development and pathogenesis of several human diseases. During stress-mediated responses caused by infections or tissue injuries, damage-associated proteins from the S100 protein family are released locally [1–3]. S100 proteins play a critical role in regulating numerous cellular processes such as enzymatic activities, cell growth, survival and differentiation. Two prominent members of the S100 protein family constitute the heterodimer calprotectin [4], S100A8 (Myeloid Related Protein, MRP8) and S100A9 (MRP14), which have been shown to play a major role during inflammatory conditions [5] including several autoimmune diseases, atherosclerosis, neurodegenerative disorders, various types of cancer and pulmonary pathologies [5–10]. They act as damage-associated molecular patterns (DAMPs) via a toll-like receptor 4 (TLR4) or receptor for advanced glycation end products (RAGE)-dependent mechanism [11,12] and thereby amplify acute and chronic inflammatory immune responses [1,12]. However, there are also reports showing an anti-inflammatory or protective role of these proteins during infection and inflammation and there is an ongoing controversy on whether they are pathogenic or rather protective. Large quantities of S100A8/S100A9 are found in the cytosol of neutrophils, which represent the most abundant proteins reaching up to 40% of the total cytosol protein content [13]. During infection, tissue damage, neutrophil extracellular trap formations (NETs) [14], and cellular necrosis, the levels of S100A8 and S100A9 are increasingly expressed in circulating neutrophils and monocytes [2] and can also be secreted passively [1,2,15].

In the past neutrophils have been shown to mediate protection against parasitic filarial nematodes. These filariae represent a huge global health problem in numerous tropical and subtropical areas and are responsible for several devastating diseases such as lymphatic filariasis (elephantiasis) or onchocerciasis (river blindness) [16,17]. Parasitic helminths modulate the host immune system to their benefit by dampening type 1 immune responses and inducing a type 2 immune shift [18,19]. Type 2 immune responses during a helminth infection are characterized by elevated IgE levels, increased production of type 2 cytokines (e.g. IL-4, IL-5, IL-13) and an expansion of eosinophils [20–22] and ILC2s [23]. Furthermore, helminths establish a regulatory, anti-inflammatory milieu by inducing regulatory cytokines (TGFβ, IL-10), increasing frequencies of regulatory T cells [24] and alternatively activated macrophages [25]. This immunomodulation enables the long-term survival of helminths in their host.

The rodent filarial nematode *Litomosoides sigmodontis* has essentially helped to improve our understanding of the protective immune responses during filarial infection, the modulation of the host’s immune system [25–29] and the impact on bystander immune responses [27,30–37].

Through the bite of the tropical rat mite vector (*Ornithonyssus bacoti*), infective *L*. *sigmodontis* L3 larvae are transmitted into the murine host’s skin and migrate via the lymphatic system, reaching the pulmonary blood circulation, transiting the lung and entering the thoracic cavity, where the filariae finally reside (2–6 days after infection (dpi) in BALB/c mice) [38]. Within the thoracic cavity, L3 larvae molt into L4 larvae stages (8-12dpi) and subsequently into adult worms (~30dpi). Starting from 50dpi, fertilized female worms release their progeny (microfilariae) that enters the peripheral blood system of BALB/c mice to complete the filarial life cycle.

During these investigations type 1 [39–42] and type 2 immune responses have been identified as essential for protection against *L*. *sigmodontis*. As for type 2 immune responses the absence of IL-4 [43,44], IL-5 [45–47], IL-6 [48], eosinophil products such as eosinophil peroxidase (EPO), major basic protein (MBP) [49] and eosinophil-specific chemoattractant eotaxin 1 (CCL11) [50] led to an increased susceptibility to *L*. *sigmodontis* infection and increased the adult worm burden and/or microfilaremia [51]. As for type 1 immune responses, Saeftel *et al*. demonstrated that the deficiency of IFN-γ led to an increased worm burden due to reduced neutrophil recruitment and encapsulation of adult worms compared to wildtype (WT) mice [42]. Neutrophils were subsequently shown in a line of experiments to be essential for protective immune responses against invading *L*. *sigmodontis* L3 larvae [40,41] as well as L3 larvae of other filarial nematodes [52]. Porthouse *et al*. showed that migrating *Brugia pahangi* third-stage larvae induce neutrophil accumulation around the invading larvae in the skin with increased associated inflammatory cytokines such as IL-6 and TNF [52]. Accordingly, delayed recruitment of neutrophils to the site of *L*. *sigmodontis* L3 infection in the skin of NOD2^-/-^ and IL-6^-/-^ mice as well as neutrophil depletion in WT animals increased the worm recovery, indicating that neutrophils are essential for protective immune responses against invading L3 larvae [40,48]. Accordingly, Cxcr4^+/1013^ mice, which have an increased number of neutrophils in the skin, reduced the *L*. *sigmodontis* worm recovery in a neutrophil-dependent manner [41].

Recently, it was demonstrated that *L*. *sigmodontis* L3 larvae trigger the expression of S100A9 in neutrophils during their migration through the lung into the thoracic cavity [38]. In the present study we investigated the impact of calprotectin during *L*. *sigmodontis* infection in C57BL/6 mice lacking S100A9 and compared the worm burden and immune responses to C57BL/6 WT controls. Of note, S100A9^-/-^ mice are also deficient of S100A8 on the protein level representing a functional double knockout mouse [53,54]. Therefore, we are refering to S100A8/S100A9 throughout the manuscript.

Our data indicates an anti-inflammatory role of S100A8/S100A9 on neutrophil activation, which reduced inflammatory responses and facilitated the migration of infective L3 larvae into the thoracic cavity.

## Methods

### Ethics statement

Animal housing conditions and animal experiments were approved by the local authorities (Landesamt für Natur, Umwelt und Verbraucherschutz Nordrhein-Westfalen register no. 87–51.04.2011A025/01; 84–02.04.2014.A327) as well as the ethical committee of the MNHN (Comité Cuvier, Licence: 68–002) and by the DDCSPP (C75-05-15). Experiments were performed according to the Directive 2010/63/EU guidelines and the relevant national legislation (French Decret 2013, 1^er^ fevrier 2013, Ministère de l'Agriculture, de l'Agroalimentaire et de la Foret). Animal welfare was scored on a scale from A-C for symptoms considering appearance, injuries, weight loss, and behaviour. A score of A required daily observations of the symptoms, a score of B, consultation of the project leader or a veterinary, and a score of C the immediate euthanasia of the affected animal. For euthanasia, animals were exposed to an overdose of isoflurane.

### Mice and infection

C57BL/6JS100A8/A9^-/-^ mice (provided by Prof. Dr. Thomas Vogl; Institute of Immunology, University Hospital of Münster, Germany) and corresponding C57BL/6J WT controls (Janvier) were bred and housed in individually ventilated cages on a 12 hour light/dark cycle at the animal facility of the Institute for Medical Microbiology, Immunology and Parasitology, University Hospital Bonn, Germany. Mice had access to food and water *ad libitum*. Only female mice were included in the experiments and mice were infected at 8–10 weeks of age with *L*. *sigmodontis* via natural infection with the intermediate host *Ornithonyssus bacoti*. Mice were exposed simultaneously to the same batch of *O*. *bacoti* mites to ensure a comparable infection of both groups [47]. For subcutaneous or intravenous infections of mice, L3 larvae were isolated from mites [38] or 5 day-infected gerbils (*Meriones unguiculatus*) [55] and 40 L3 larvae were subcutaneously injected into the neck region or inguinal regions or intravenously into the caudal vein. Necropsy was performed on day 12 after *L*. *sigmodontis* infection using an overdose of Isoflurane (Forene) and worms were isolated from the thoracic cavity by lavage and quantified using a binocular microscope.

### Isolation of spleen, thoracic cavity, bronchoalveolar lavage and skin cells

Mice were euthanized per inhalation of an overdose of Isoflurane. In order to isolate thoracic cavity cells the thoracic cavity was initially flushed with 1ml PBS (Gibco) and the lavage centrifuged at 400g for 5min at 4°C. The supernatant was frozen at -20°C for further cytokine and chemokine measurements. The separated cells were combined with the cells gathered during a second lavage with 9ml PBS.

To assess bronchoalveolar cells, the trachea of euthanized mice was exposed and a venous catheter (Vasofix Braunüle, Braun) was inserted at the cervico-thoracic junction. The bronchoalveolar space was washed with 10ml PBS and the cell isolation was performed as described above.

Spleens of dissected mice were removed after necropsy, homogenized and pressed through a 70μm cell strainer. Cells were centrifuged at 400g for 10min at 4°C, the supernatant was discarded and red blood lysis was performed with 1ml of RBC lysis buffer (ThermoFisher) for 5min at room temperature. Lysis was stopped by adding 9ml of RPMI 1640 medium (Gibco). After centrifugation the supernatant was removed and the pellet was resuspended in 10ml of RPMI 1640 media. Thoracic cavity, bronchoalveolar lavage (BAL) and spleen cell numbers were determined by Casy Cell Counter (Roche) and prepared for flow cytometry or cell culture stimulation experiments.

For skin analyses, mice were shaved 48h prior injection on the upper hind leg region. 10 infective L3 larvae isolated from infected gerbils by pleural lavage [55] were suspended in 50μl pre-warmed RPMI 1640 media and injected intradermally in the centre of the shaved area. PBS was used as corresponding control. 3h after injection, skin biopsies around the injection site (1cm^2^) were taken and incubated with liberase (containing DNase (Thermofisher)) for 50min at 37°C. Digested tissue was filtered through a 70μm strainer (Milteny). RBC lysis was performed with 1ml of RBC lysis buffer as described above and subsequently the cell suspension was washed with PBS. Cell numbers were determined by Casy Cell Counter and cells were subsequently prepared for flow cytometric analysis.

### Preparation of *L*. *sigmodontis* adult worm extract

Adult worms, freshly isolated from the thoracic cavities of infected gerbils were scavenged in endotoxin-free PBS, before being homogenized using a mortar under sterile conditions. After centrifugation at 300g for 10min at 4°C, the supernatant was collected and protein concentration of the crude extract (LsAg) was determined using the Advanced Protein Assay (Cytoskeleton) [56,57].

### Spleen cell culture

2x10^6^ cells/ml of isolated splenocytes were plated in enriched media (RPMI 1640, 10% FCS, 4mM- L-glutamine, 100U/ml penicillin, 100μg/ml streptomycin) (all purchased from PAA Laboratories). Isolated cells remained either unstimulated or were stimulated with 2.5μg/ml Concanavalin A (Sigma) or with 25μg/ml *L*. *sigmodontis* adult worm extract (LsAg). Cells were cultured at 37°C, 5% CO_2_ and 90% humidity for 72h and then centrifuged at 400g for 5min. Cell culture supernatants were taken for cytokine measurements and stored at -20°C.

### Measurement of cytokines, chemokines and enzymes by ELISA

Measurements were performed from the first ml of thoracic cavity lavage, BAL and splenocyte culture supernatants by ELISA. IFN-γ and TNF (ThermoFisher) as well as CXCL-1, CXCL-2, CXCL-5, elastase, myeloperoxidase and S100A8/A9 (all R&D) were measured according to manufacturers’ guidelines.

### Flow cytometry

Thoracic cavity, spleen, BAL and skin cells were analyzed by flow cytometry. Cells were isolated as described above and subsequently blocked with PBS/1% BSA including 0.1% rat IgG (Sigma) and stained. Flow cytometric analysis was performed using a combination of the following surface markers: CD4-FITC, CD8-APC, SiglecF-PE, F4/80-PerCP-Cy5.5, CD11b-APC-Cy7 (all BioLegend), and Ly6G-PE-Cy7 (ThermoFisher). Thoracic cavity and spleen CD4^+^ T cells and CD8^+^ T cells were identified as CD4^high^ or CD8^high^ cells, respectively; neutrophils as Ly6G^high^, CD4^low^; eosinophils as SiglecF^high^, F4/80^low^; macrophage populations were identified as F4/80^high^, SiglecF^low^. BAL macrophages were identified as F4/80^high^, SiglecF^high^ and neutrophils as Ly6G^high^/CD11b^high^. Cells were further analyzed with MHCII-PE (BioLegend) in a different panel. Cells were stained and after another wash and centrifugation step cells were analysed by flow cytometry. Analysis was performed using a BD FACS Canto system and data was subsequently analyzed using the FACS Diva 5.1 software (BD Biosciences). During analysis, cut-offs were set using the fluorescence minus one (FMO) approach. The gating strategy is shown in S1 Fig.

### Neutrophil purification

Experimental animals were euthanized per inhalation of an overdose of Isoflurane. Femurs of S100A8/A9^-/-^ and WT controls were removed surgically and remaining tissue was detached. The condyles of the bones were cut and subsequently the bone marrow was flushed with 5ml of cold sterile PBS using a syringe. The flow through was filtered (70μm strainer) and after a centrifugation step at 400g for 5min at 4°C the supernatant was discarded and red blood lysis was performed by adding 1ml RBC lysis buffer for 5min. Lysis was stopped by adding 9ml of cold, sterile PBS. Cells were centrifuged again at 400g for 5min at 4°C and the pellet was resuspended in 3ml of PBS. Murine bone marrow-derived neutrophils were purified by a 3-layer Percoll gradient method using 72%, 64% and 52% Percoll solutions in RPMI 1640 with the cells layered on top. After centrifugation at 1500g for 30min without brake, neutrophils were recovered from the interface between the 72% and 64% layers. Cells were washed with PBS and centrifuged at 400g for 5min. Next, the supernatant was removed and cells were resuspended in 1ml of PBS. Cell numbers were determined by Casy Cell Counter and used for flow cytometric analysis or cell culture stimulation experiments.

### Neutrophil cell culture

2x10^5^ WT and S100A8/A9^-/-^ bone marrow-derived neutrophils were cultured in a total volume of 200μl of RPMI 1640 media containing 10% FCS, 1% L-Glutamine, 100U/mL Penicillin, 100μg/ml Streptomycin for 7h at 37°C (5% CO_2,_ 90% humidity) in triplicates. Quantification of apoptosis (FITC Annexin V) and necrosis (Propidium Iodide) was performed according to manufacturer’s protocols (BD). The supernatant was collected and frozen at -20°C for subsequent cytokine and chemokine analysis and cells were used for further flow cytometric analysis.

### Neutrophil depletion

Neutrophil depletion was performed 18h prior to natural *L*. *sigmodontis* infection in S100A8/A9^-/-^ and corresponding WT mice by intranasal treatment with 250μg anti-Ly6G antibody (Clone 1A8, BioXCell) in 50μl pre-warmed sterile PBS. Treatment was repeated every second day until 5 days after *L*. *sigmodontis* infection. Corresponding control mice were treated with an IgG2a isotype antibody (Clone C1.18.4, BioXCell). Neutrophil depletion within the lung was confirmed in a pilot experiment, where IgG2a isotype control and anti-Ly6G depletion antibody were given every second day with a total of three injections and confirmation of the depletion 24h after the last injection. The systemic effect of intranasal neutrophil depletion was tested within the blood, pleura, spleen, lung as well as skin in naïve mice, which were treated trice (every second day) either with the isotype control or with the neutrophil depleting anti-Ly6G antibody (250μg). Results are shown in S2 Fig.

### *L*. *sigmodontis* L3 larvae *in vitro* assay

For motility experiments, 1x10^5^ WT and S100A8/A9^-/-^ neutrophils were cultured in triplicates in 200μl enriched media (RPMI 1640 media + 10% FCS, 1% L-Glutamine, 100U/mL Penicillin, 100μg/ml Streptomycin) for 48h at 37°C (5% CO_2,_ 90% humidity) with 10 L3 larvae. Motility inhibition was assessed daily by a five point scoring system; Score 4: vigorous, fidgeting movements. Score 3: slightly impaired vigorous movements. Score 2: impaired motility/slow movements. Score 1: mostly immobile/single movements. Score 0: fully immobile within 20s observation period.

### Lung histology and immunohistology

After necropsy, the lung was filled with 4% formalin-PBS (Sigma Aldrich) and fixed overnight. Afterwards, the fixative was renewed for another 24h, before it was replaced by 70% ethanol for 2–7 days prior paraffin embedding. Five-micron-thick serial sections were prepared. Lungs were stained with 1) hematoxylin-eosin to analyse the general structure of the lung; 2) Congo red to visualise eosinophils following the online protocol http://www.ihcworld.com/_protocols/special_stains/congo_red_highman.htm; 3) Alcian Blue/Periodic Acid Schiff (AB-PAS) staining to visualize mucus producing cells using the protocol: http://www.ihcworld.com/_protocols/special_stains/alcian_blue_pas_ellis.htm; 4) a Ly6G/Ly6C immunostaining to visualize neutrophils. Briefly, sections were washed, deparaffinized, rehydrated, and rinsed in PBS. A proteinase K solution (0.004%), diluted in a 1:1 glycerol-modified Tris Buffer (EDTA 3.7%, Triton X-100 0.5%, pH 8), was used for antigen retrieval for 10min at 37°C, then tissue endogenous peroxidases were blocked by utilizing a dual endogenous enzyme block (Dako) and biotin/avidin by a biotin blocking kit (Vector). Neutrophils were stained in a 1/200 dilution with a primary antibody against Ly6G/Ly6C (rat monoclonal AB, clone NIMP-R14, Hycult Biotech) dissolved in blocking serum (Vectastain kit, Vector). Detection was conducted by the Vectastain Elite ABC kit (Vector) and revelation was performed with a high sensitivity AEC substrate (Dako), which was subsequently counterstained with a Mayer’s hematoxylin solution (Merck).

### Vascular permeability assay

Mice were anaesthetized with Rompun (2%; Bayer) and Ketamine (50mg/mL; Ratiopharm) and *in vivo* vascular permeability was determined via the intracutaneous injection of 10μg LsAg in one ear and PBS as corresponding control in the other ear. After 3min, mice were injected i.v. with NaCl 0.9% containing 0.9mg of Evans Blue (Sigma-Aldrich). After 10min, mice were euthanized by cervical dislocation. Ears were removed and transferred into 500μl of >99% formamide (Sigma-Aldrich) and incubated at 56°C for 24h. Evans Blue leakage was then determined by optical density at 620nm (Spectramax 240 pc, Molecular Devices).

### Statistics

Statistical analyses were performed with GraphPad Prism software Version 5.03 (GraphPad Software). Normal distribution of the data was tested with D’agostino test. Non-parametrically distributed data and data of non-sufficient animal numbers for parametric testing were analyzed by Kruskal-Wallis test followed by Dunn’s posthoc test. Differences between two unpaired groups were tested for statistical significance with the Mann-Whitney-U-test. P-values <0.05 were considered statistically significant. Power analysis with G*Power (3.1.9.4) indicated that a sample size of 8 mice per group provides a power >95% and an α-error of 0.05. Data from pooled experiments were tested by two-way ANOVA and Spearman’s test for heteroscedasticity using GraphPad Prism software Version 8. Only experiments that did not pass the heteroscedasticity test were pooled.

## Results

### Decreased worm burden upon natural and subcutaneous infection in S100A8/A9^-/-^ mice

In order to investigate the impact of S100A8/A9 during filarial infection, WT and S100A8/A9^-/-^ mice were subcutaneously infected with 40 *L*. *sigmodontis* L3 larvae. Concentration of calprotectin within the thoracic cavity lavage was analysed between naïve and infected WT mice and necropsy of WT and S100A8/A9^-/-^ *L*. *sigmodontis*-infected animals was performed at 12dpi, a time-point where all L3 larvae reached the thoracic cavity and molted into L4 larvae. Following necropsy, analysis of the worm burden, cellular composition, neutrophil-associated chemokines, and neutrophil elastase were performed.

**Fig 1:**
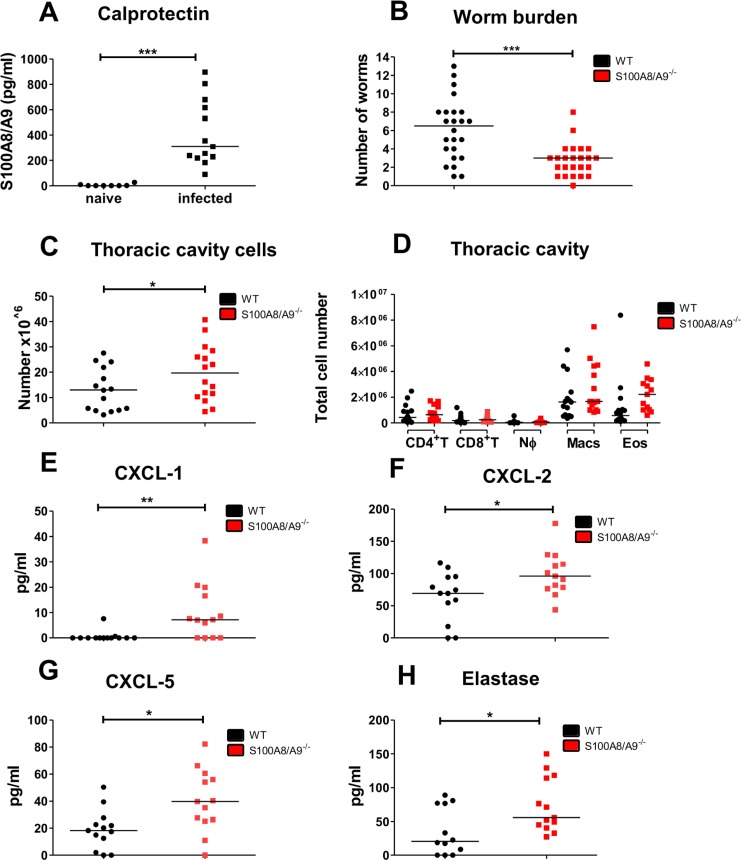
Reduced worm burden, but increased neutrophil mediator release during *L*. *sigmodontis* infection in S100A8/A9^-/-^ mice. Concentration of calprotectin (**A**) and worm burden following subcutaneous *L*. *sigmodontis* infection in WT and S100A8/A9^-/-^ mice at 12 days after infection (**B**). Number of thoracic cavity cells (**C**) and total thoracic cavity cell counts of CD4+ T cells (CD4^+^ T), CD8+ T cells (CD8^+^ T), neutrophils (NØ), macrophages (Macs) and eosinophils (Eos) (**D**) 12 days after *L*. *sigmodontis* infection in WT and S100A8/A9^-/-^ mice. Concentrations of CXCL-1 (**E**), CXCL-2 (**F**), CXCL-5 (**G**), and elastase (**H**) in the thoracic cavity lavage 12 days after *L*. *sigmodontis* infection in WT and S100A8/A9^-/-^ mice. Results are shown as median (**A**-**H)**. Statistical significance was analyzed by two-tailed non-parametric Mann-Whitney-U-test. *p<0.05, **p<0.01, ***p<0.001. Data shown are pooled of two (**A**, **C**-**H**) and three independent experiments (**B**) with 4–8 mice per group.

In accordance to Karadjian and colleagues [38], *L*. *sigmodontis* infection led to a significant increase of S100A8/A9 levels within the thoracic cavity lavage of infected WT animals compared to naïve WT mice (Fig 1A). Subcutaneous *L*. *sigmodontis* infection in S100A8/A9^-/-^ mice resulted in a significantly reduced worm burden compared to WT infected animals (Fig 1B). The reduced number of worms was associated with a significant increase in thoracic cavity cells in the S100A8/A9^-/-^ mice (Fig 1C), while total cell counts of CD4^+^ T cells, CD8^+^ T cells, neutrophils, macrophages, and eosinophils in the thoracic cavity were not different between the two mouse strains (Fig 1D). However, levels of neutrophil-associated chemokines, i.e. CXCL-1 (Fig 1E), CXCL-2 (Fig 1F), CXCL-5 (Fig 1G), and elastase (Fig 1H) were significantly increased in the thoracic cavity lavage of S100A8/A9^-/-^ mice compared to WT controls. Similarly, absolute spleen cell numbers were significantly increased in the S100A8/A9^-/-^ mice in comparison to WT controls at 12dpi (S3A Fig) with significant increases of CD4^+^ T cells, CD8^+^ T cells and neutrophil numbers in the spleens of S100A8/A9^-/-^ mice in comparison to WT controls (S3B Fig). Interestingly, splenocytes of S100A8/A9^-/-^ mice cultured in the presence of LsAg exhibited an increased production of IFN-γ (S3C Fig) and TNF (S3D Fig) compared to splenocytes of WTmice.

To address whether a similar phenotype is obtained in S100A8/A9^-/-^ mice after natural infection, S100A8/A9^-/-^ mice and WT controls were infected with *L*. *sigmodontis* via the tropical rat mite (*Ornithonyssus bacoti*). In comparison to the subcutaneous infection, mite-transmitted L3 larvae have to additionally bypass the epidermis containing Langerhans cells and dermis-containing dendritic cells, macrophages and T cells. Nevertheless, natural *L*. *sigmodontis* infection led to similar results as observed during subcutaneous *L sigmodontis* infection, showing a significantly reduced worm burden (S4A Fig) and an increased thoracic cavity cell count (S4B Fig) in S100A8/A9^-/-^ mice compared to WT controls. Total cell counts of CD4^+^ T cells, CD8^+^ T cells, neutrophils, macrophages and eosinophils (S4C Fig) were comparable between WT and S100A8/A9^-/-^ mice. Similar to the subcutaneous infection, concentrations of CXCL-1 (S4D Fig), CXCL-2 (S4E Fig), CXCL-5 (S4F Fig), and elastase (S4G Fig) were significantly increased in S100A8/A9^-/-^ animals compared to WT controls after natural *L*. *sigmodontis* infection.

To further investigate the impact of S100A8/A9 on *L*. *sigmodontis* infection at a later time point of infection, animals were analysed at 29dpi, a time point where the molting into adult male and female worms has occurred. Similar to the results obtained from 12dpi, adult worm counts were significantly decreased in S100A8/A9^-/-^ mice compared to WT controls at 29dpi (S5A Fig). Thoracic cavity cell numbers were still significantly elevated in S100A8/A9^-/-^ mice compared to WT mice (S5B Fig), while total cell counts of eosinophils, neutrophils and macrophages were comparable between S100A8/A9^-/-^ mice and WT controls (S5C Fig).

In conclusion these results indicate that S100A8/A9 may be involved in anti-inflammatory immune responses during *L*. *sigmodontis* infection, since S100A8/A9^-/-^ mice were characterized by an increased number of thoracic cavity cells and elevated concentrations of neutrophil-associated chemokines such as CXCL-1, CXCL-2, CXCL-5, and elastase, which may contribute to the elimination of *L*. *sigmodontis* in S100A8/A9^-/-^ mice.

### Increased cellular immune responses in S100A8/A9^-/-^ mice following intradermal L3 larvae injection

**Fig 2:**
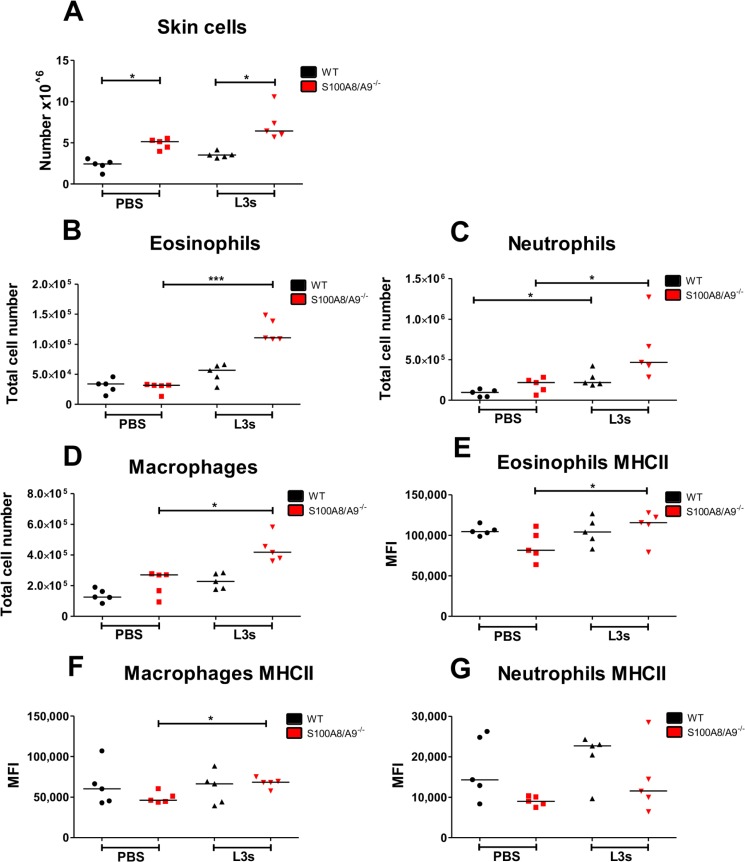
Intradermal injection of infective *L*. *sigmodontis* L3 larvae leads to increased cellular immune responses within the skin of S100A8/A9^-/-^ mice. Total number of skin cells (**A**), eosinophils (**B**), neutrophils (**C**), and macrophages (**D**) and their respective MHCII expression (**E**, **F**, **G**) in WT and S100A8/A9^-/-^ mice 3h after intradermal *L*. *sigmodontis* L3 or PBS injection. Results are shown as median. Statistical significance of not normally distributed data (**A**-**G**) was analyzed by Kruskal-Wallis followed by Dunn’s multiple comparison test. p<0.05, ***p<0.001. Representative data from one out of two independent experiments with 5 mice per group (**A-G**).

Previous studies from our lab indicate that the skin represents the first barrier invading L3 larvae have to pass and a subcutaneous injection of L3 larvae circumvents some of those protective immune responses in the skin. Accordingly, increased worm burden in IL-6 and NOD2 deficient mice, which showed a delayed neutrophil recruitment to the site of infection in the skin, were overcome by subcutaneous infection [40,48], resulting in a worm burden that was comparable to immunocompetent WT animals. Given the decreased worm burden in S100A8/A9^-/-^ mice that was also present after subcutaneous infection, which bypasses the epidermis and dermis, we next assessed S100A8/A9-dependent immunological changes within the skin 3h after intradermal injection of mice with 10 L3 larvae. S100A8/A9^-/-^ mice had a higher total cell count after intradermal injection of PBS and L3 larvae compared to WT controls (Fig 2A). L3 inoculation in S100A8/A9^-/-^ mice resulted in a significantly increased total cell number of eosinophils (Fig 2B), neutrophils (Fig 2C) and macrophages (Fig 2D) in comparison to PBS-injected S100A8/A9^-/-^ mice. L3-injected WT mice showed also an elevated total cell count of neutrophils compared to PBS-treated WT animals (Fig 2C). Although no statistical significant differences were obtained in the total number of eosinophils, neutrophils and macrophages between WT and S100A8/A9^-/-^ mice following L3 injection, by trend S100A8/A9^-/-^ mice had the highest cell counts of these cell populations (Fig 2B–2D). Regarding cell activation, L3 injection led to an increased MHCII expression on eosinophils (Fig 2E) and macrophages (Fig 2F) in the skin of S100A8/A9^-/-^ mice compared to cells of PBS-treated S100A8/A9^-/-^ animals, while there was a trend for a lower MHCII expression on neutrophils (Fig 2G) of S100A8/A9^-/-^ animals in comparison to WT controls independent on PBS or L3 injection. The results from the repeat experiment are shown in S6 Fig and confirmed the above described results.

Based on these results cellular immune responses against invading L3 larvae are increased within the skin of S100A8/A9^-/-^animals. Thus, they may contribute to enhanced clearance of L3 larvae in S100A8/A9^-/-^ animals even after subcutaneous injection of L3 larvae and circumvention of the epidermis and dermal layers.

### S100A8/A9 deficiency increases inflammatory responses within the lung

In the *L*. *sigmodontis* filarial mouse model, infective L3 larvae are inoculated into the skin of the host by the bite of blood-feeding mites and migrate via the lymphatic, then pulmonary blood system into the thoracic cavity, the site of adult worm residence. At 12dpi, a time point L3 larvae have reached the thoracic cavity and molted into the L4 stage, a vascular lung pathology was observed (S7A Fig) compared to naive C57BL/6 mice (S7B Fig). Lung pathology caused by migrating L3 included an increase of congested blood vessels with cellular infiltrates in perivascular spaces (PVS) surrounding blood vessels (S7C and S7E Fig) compared to lungs of naïve WT controls (S7D Fig). Some blood vessels were not congested and presented low cellular infiltrates in the PVS, but adjacent bronchial epithelium was positive for Periodic Acid-Schiff (PAS; bright pink staining), a signature of mucus production by epithelial cells (S7C Fig lower panel). *L*. *sigmodontis* infection was furthermore associated with an increased number of bronchoalveolar goblet cells (S7F Fig).

Lung pathology in S100A8/A9^-/-^ mice contained by trend less inflamed/congested blood vessels (p = 0.14; S7E Fig) and less bronchi with goblet cells in comparison to infected WT animals (p = 0.22; S7F Fig). Filarial worm burden at 12dpi correlated moderate-positively with the number of congested blood vessels and bronchi with goblet cells (S7G Fig; r = 0.66; p = 0.001, S7H Fig; r = 0.68; p = 0.001). These results indicate that the observed lung pathology may be caused by migrating L3 larvae. Analysis of PVS cellular content further showed a significant influx of cells especially in congested blood vessels of infected WT and S100A8/A9^-/-^ mice, reaching statistical significance for infected S100A8/A9^-/-^ animals (p<0.05; S7I Fig).

Furthermore, no influx of neutrophils were observed in the PVS of infected animals at 12dpi surrounding congested (S7J Fig) and intact blood vessels (S7K Fig), but an increased number of eosinophils was present (S7L Fig, S7M Fig). However, neutrophil infiltrates have recently been observed at an earlier time point of *L*. *sigmodontis* infection in pulmonary capillaries (6h) and in the PVS (4dpi) upon migration of L3 larvae and were associated with an increased expression of S100A9 [38].

**Fig 3:**
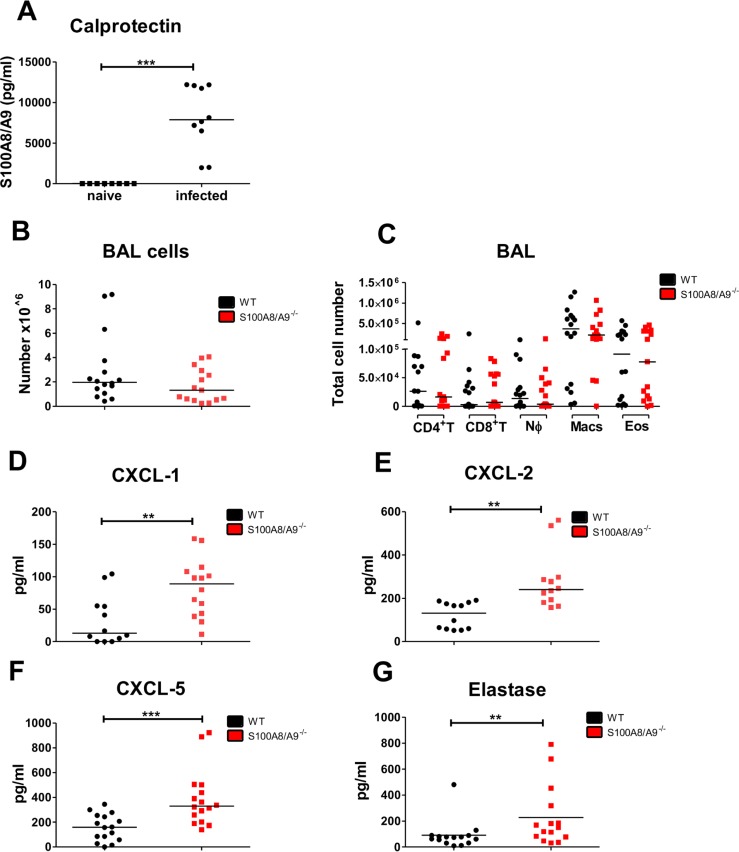
Increased immune responses to migrating *L*. *sigmodontis* L3 larvae within the BAL of S100A8/A9^-/-^ mice. Concentration of calprotectin **(A)** and total number of bronchoalveolar cells (**B**) as well as CD4+ T cell (CD4^+^ T), CD8+ T cell (CD8^+^ T), neutrophil (NØ), macrophage (Macs), and eosinophil (Eos) cell counts (**C**) in WT and S100A8/A9^-/-^ mice 12 days after subcutaneous *L*. *sigmodontis* infection. Concentrations of CXCL-1 (**D**), CXCL-2 (**E**), CXCL-5 (**F**), and elastase (**G**) in the bronchoalveolar lavage 12 days after *L*. *sigmodontis* infection of WT and S100A8/A9^-/-^ mice. Results are shown as median (**A-G**). Statistical significance was analyzed by two-tailed Mann-Whitney-U-test (**A-G**). **p<0.01., ***p<0.001. Data shown in **A-G** are pooled of 2–3 independent experiments with 5–8 mice per group.

To elucidate how S100A8/A9 deficiency may impact immune responses during the migration of *L*. *sigmodontis* L3 larvae within the lung, concentration of S100A8/A9 in naïve and infected WT mice as well as absolute bronchoalveolar lavage (BAL) cell numbers and the cellular composition of the BAL in *L*. *sigmodontis*-infected WT and S100A8/A9^-/-^ mice were determined. *L*. *sigmodontis* infection led to a significant increase of S100A8/A9 within the BAL compared to naïve WT controls (Fig 3A). Analysis of the cellular composition within the BAL following subcutaneous infection showed no differences for total BAL cell counts as well as CD4^+^ T cell, CD8^+^ T cell, neutrophil, macrophage, and eosinophil BAL counts between S100A8/A9^-/-^ and WT mice (Fig 3B and 3C). Analysis of the chemokine response in the BAL led to similar results as observed in the thoracic cavity lavage with S100A8/A9^-/-^ mice having significantly increased levels of CXCL-1 (Fig 3D), CXCL-2 (Fig 3E), CXCL-5 (Fig 3F), and elastase (Fig 3G).

Taken together, these findings indicate that migrating L3 larvae trigger pulmonary inflammation and that the absence of S100A8/A9 led to a pronounced chemokine response within the BAL.

### Increased activation of S100A8/A9^-/-^ neutrophils

**Fig 4:**
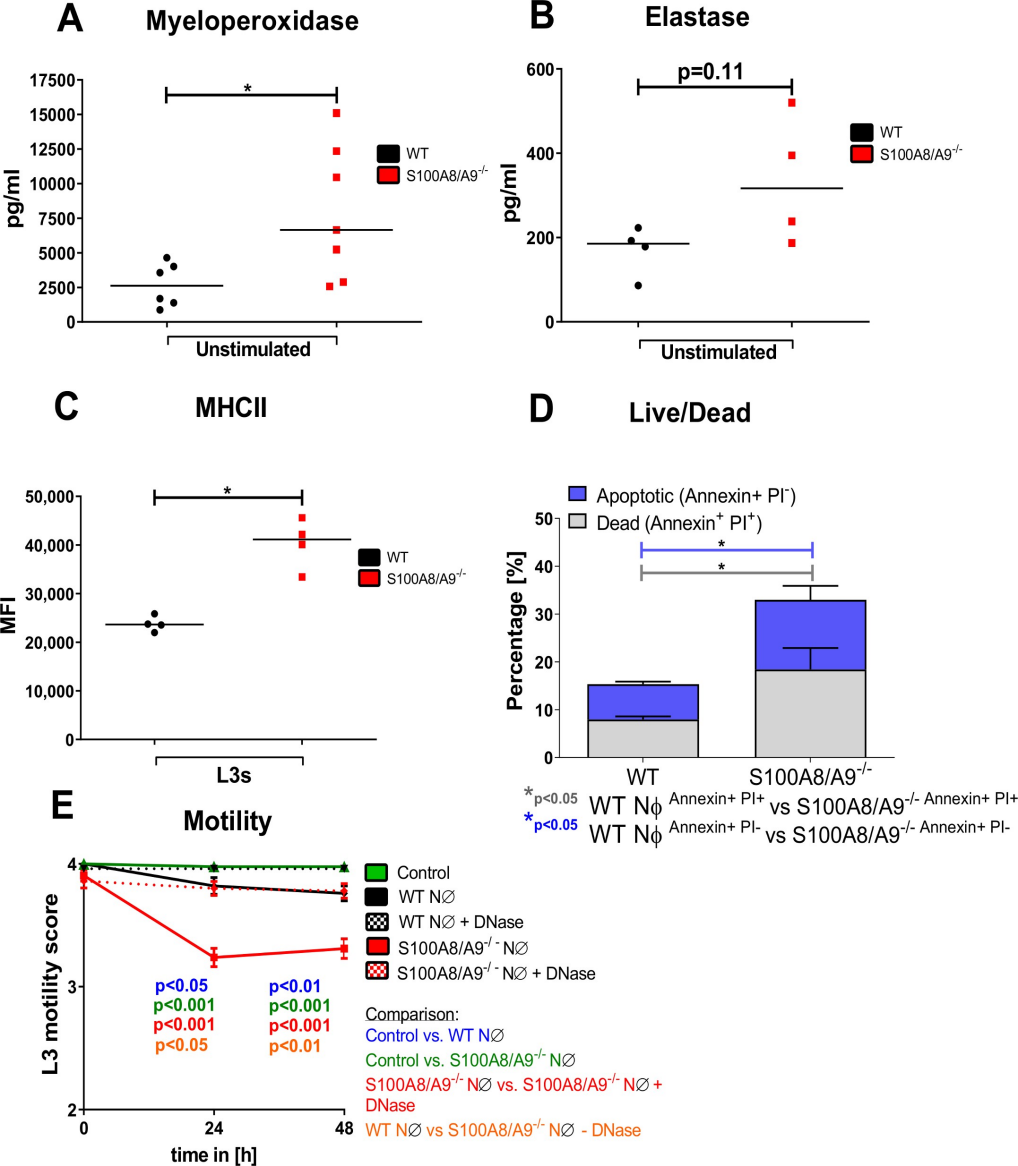
Increased activation and apoptosis of S100A8/A9^-/-^ neutrophils. Baseline Myeloperoxidase concentration (**A**), elastase concentration (**B**) and L3 larval-induced MHCII expression (**C**) of *in vitro* cultured bone marrow-derived-WT and S100A8/A9^-/-^ neutrophils (NØ). Apoptosis (**D**) of bone marrow-derived neutrophils from WT and S100A8/A9^-/-^ mice after 7h of *in vitro* culture without stimulation. Motility score of L3 larvae cultured in the presence of WT or S100A8/A9^-/-^ neutrophils with or without DNase (**E**). Results are shown as median (**A-C**), mean and SEM (**D**, **E**). Statistical significance was analyzed by two-tailed non-parametric Mann-Whitney-U-test (**A-D**) and two-way ANOVA followed by Bonferroni’s post-hoc test (**E**). *p<0.05. Data shown are pooled of 2 independent experiments with 2–4 mice per group.

Based on the increased production of neutrophil-associated chemokines in the BAL and thoracic cavity of S100A8/A9^-/-^, we compared the neutrophil activation of S100A8/A9^-/-^ and WT mice. Myeloperoxidase (MPO) as well as elastase, enzymes which are abundantly expressed in neutrophils, were increasingly released (MPO: p<0.05; elastase: p = 0.11) from bone marrow-derived neutrophils of S100A8/A9^-/-^ mice compared to WT neutrophils at baseline conditions *in vitro* (Fig 4A and 4B). Similarly, bone marrow-derived neutrophils of S100A8/A9^-/-^ mice cultured in the presence of L3 larvae exhibited an increased MHCII expression compared to neutrophils of WT mice (Fig 4C). This increased activation of S100A8/A9^-/-^ neutrophils was further associated with an increased apoptosis, resulting in a significantly higher frequency of apoptotic and dead neutrophils (Fig 4D). To test whether changes in neutrophil activation impact L3 larvae motility, L3 larvae were cultured in the presence of neutrophils isolated from WT and S100A8/A9^-/-^ mice. L3 larvae cultured with S100A8/A9^-/-^ neutrophils showed an earlier inhibition of L3 motility compared to L3 larvae cultured with neutrophils of WT mice (Fig 4E). Addition of DNase abrogated the inhibitory effect of S100A8/A9^-/-^ neutrophils on L3 motility, suggesting that the effect is DNA trap-dependent. In summary, these findings suggest that neutrophils from S100A8/A9^-/-^ mice exhibit an increased activation and are more potent in inhibiting the motility of *L*. *sigmodontis* L3 larvae.

### Depletion of neutrophils abrogates the S100A8/A9-mediated protective effect

**Fig 5:**
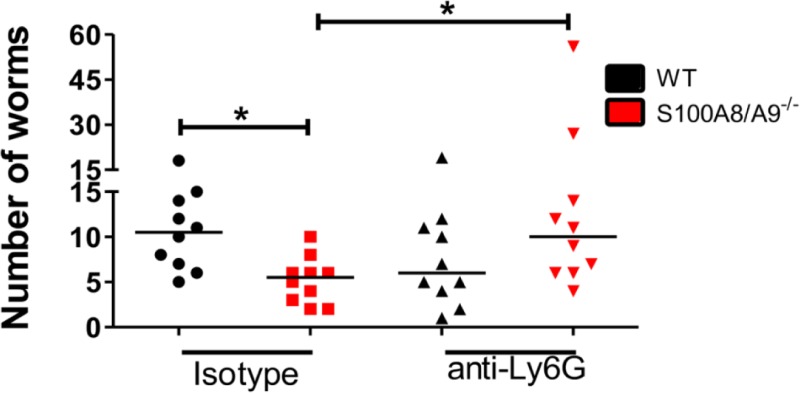
Intranasal depletion of neutrophils during the migratory phase of *L*. *sigmodontis* infection abrogates the S100A8/A9^-/—^mediated reduction in worm burden. Worm burden 12 days after *L*. *sigmodontis* infection of WT and S100A8/A9^-/-^ mice intranasally treated with anti-Ly6G or isotype control. Statistical significance of not normally distributed data was analyzed by Kruskal-Wallis followed by Dunn’s multiple comparison test. *p<0.05. Results are shown as median. The figure shows pooled data of two independent experiments with 5 mice per group and experiment.

Based on our observations that S100A8/A9^-/-^ neutrophils have an increased activation and impact on L3 motility *in vitro*, we investigated whether depletion of neutrophils using an intranasal injection of a depleting anti-Ly6G antibody during the early phase of a natural *L*. *sigmodontis* infection abrogates the protective effect on the worm burden. As expected, isotype-treated S100A8/A9^-/-^ mice had reduced worm counts compared to WT controls at 12dpi (Fig 5), confirming our previous results upon natural infection (S4A Fig). Depletion of neutrophils led to a significantly increased worm recovery in S100A8/A9^-/-^ mice compared to isotype-treated S100A8/A9^-/-^ controls, resulting in a worm burden that was comparable to isotype-treated WT controls (Fig 5). However, intranasal anti-Ly6G treatment had a systemic effect, as frequencies of neutrophils within the BAL (S2A Fig), thoracic cavity (S2B Fig), blood (S2C Fig), spleen (S2D Fig), as well as within the skin (S2E Fig) were diminished following anti-Ly6G treatment. These data indicate that neutrophils are mediating the protective effect seen during *L*. *sigmodontis* infection in S100A8/A9^-/-^ mice, although the specific location of this effect remains unclear.

### Increased worm burden in S100A8/A9^-/-^ mice upon intravenous injection of L3 larvae

**Fig 6:**
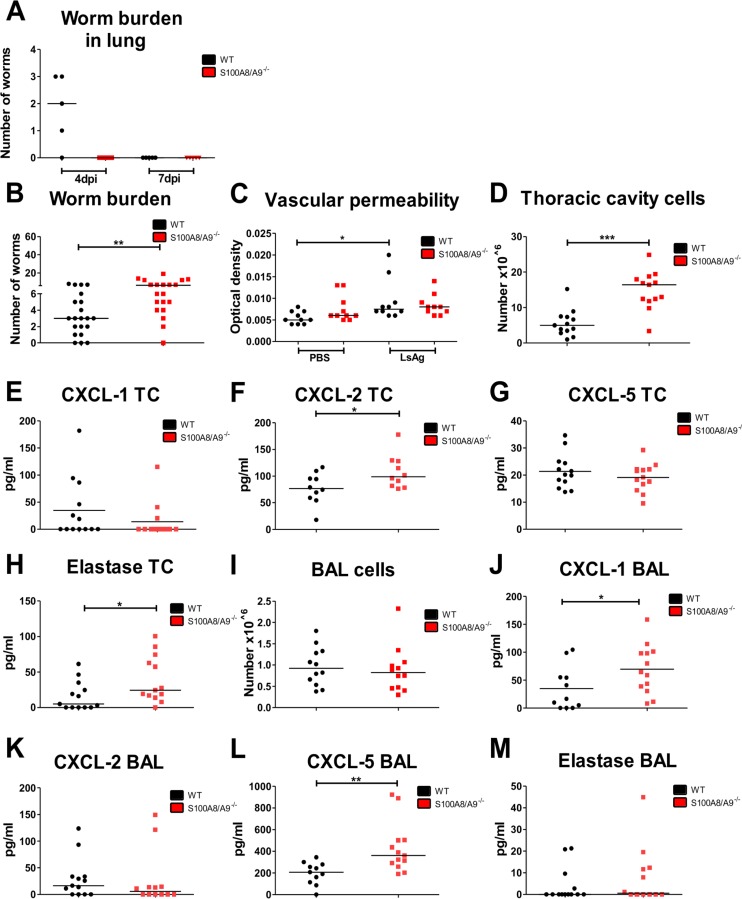
Intravenous injection leads to an increased worm burden in S100A8/A9^-/-^ mice. Total number of worms within the lung of WT and S100A8/A9^-/-^ mice 4 and 7 days after *L*. *sigmodontis* infection (**A**) as well as adult worm burden in the thoracic cavity 12 days after intravenous infection (**B**). Vascular permeability (**C**), total number of thoracic cavity cells (**D**), concentrations of CXCL-1 (**E**), CXCL-2 (**F**), CXCL-5 (**G**) and elastase (**H**) in the thoracic cavity avage (TC) as well as total number of bronchoalveolar cells (**I**) and concentrations of CXCL-1 (**J**), CXCL-2 (**K**), CXCL-5 (**L**) and elastase (**M**) in the bronchoalveolar lavage (BAL) of 12-day *L*. *sigmodontis-*infected WT and S100A8/A9^-/-^ mice. Results are shown as median (**A**-**M**). Statistical significance was analyzed by two-tailed non-parametric Mann-Whitney-U-test (**A**-**B**, **D-M**) and Kruskal-Wallis followed by Dunn’s multiple comparison test (**C**). *p<0.05, **p<0.01, ***p<0.001. Data shown in (**A**) consist of one experiment with 5 mice per group. Remaining data are pooled of three (**B**) and two independent experiments (**C**-**M**) with 5–8 mice per group.

Upon natural infection, invading L3 larvae have to pass the skin barrier, migrate via the lymphatics, enter the pulmonary blood circulation, exit the capillaries, cross the lung and enter the thoracic cavity between 2h and 6 days after infection [38]. Thus, the worm burden within the lung was assessed 4 and 7dpi. At 4dpi L3 larvae were found in the lungs of WT, but not S100A8/A9^-/-^ mice and at 7dpi no L3 larvae were found in both strains (Fig 6A). This indicates that either protective immune responses in S100A8/A9^-/-^ mice occur before the entrance into the lungs or there is an accelerated elimination of L3 larvae within the lungs in the absence of S100A8/A9.

To further address this point, S100A8/A9^-/-^ and corresponding WT mice were injected intravenously with 40 L3 larvae. During intravenous injection skin and lymphatic phases are entirely bypassed, leading to a simultaneous, accelerated entrance of the L3 larvae into the thoracic cavity [38]. Interestingly, the intravenous injection of L3 larvae resulted in an increased worm burden in S100A8/A9^-/-^ mice in comparison to WT controls (Fig 6B). One possible explanation is that the circumvention of the skin barrier and the lymphatic route leads to the increased worm burden upon intravenous infection in S100A8/A9^-/-^ mice. Therefore, we assessed the vascular permeability in S100A8/A9^-/-^ mice and WT controls, as this was previously shown to facilitate L3 migration as well [58]. Intradermal injection of filarial adult worm extract (LsAg) led to an increased permeability in WT and S100A8/A9^-/-^ animals compared to PBS controls (Fig 6C). However no statistically significant differences were observed between LsAg-treated WT and S100A8/A9^-/-^ mice, suggesting that the absence of S100A8/A9 does not impact vascular permeability.

*Per se*, intravenously-infected S100A8/A9^-/-^ mice generated immune responses in the thoracic cavity that were similarly observed following natural and subcutaneous infection, with thoracic cavity cell numbers being increased (Fig 6D) and significantly higher levels of CXCL-2 (Fig 6F) and elastase (Fig 6H) within the pleural lavage of S100A8/A9^-/-^ animals, whereas the concentrations of CXCL-1 (Fig 6E) and CXCL-5 (Fig 6G) were comparable between WT and S100A8/A9^-/-^ mice. Furthermore, no differences in the total BAL cell counts were observed between WT and S100A8/A9^-/-^ mice (Fig 6I) after intravenous *L*. *sigmodontis* infection. Levels of CXCL-1 (Fig 6J) and CXCL-5 (Fig 6L) were significantly elevated within the BAL of S100A8/A9^-/-^ mice compared to WT controls. No statistically significant differences were observed in the concentrations of CXCL-2 (Fig 6K) and elastase (Fig 6M) between WT and S100A8/A9^-/-^ animals. These results indicate that the protective effect observed in S100A8/A9^-/-^ mice is abrogated after intravenous infection resulting in an increased worm burden in the absence of S100A8/A9.

## Discussion

Modulation of the host immune system by parasitic filarial nematodes is a key mechanism to evade specific immune responses during early and chronic phases of infection. Although the inflammatory [1] and the anti-inflammatory properties [59] of the S100 calgranulin proteins have been characterized, their role during filarial infection remained elusive. As S100A8/A9 exhibits chemotactic properties on neutrophils [60] and is expressed in almost 20% of the cytosolic protein content in murine neutrophils and 45% in human neutrophils, and is increasingly expressed in lungs following *L*. *sigmodontis* L3 larvae migration [38], we investigated the impact of S100A8/A9 on neutrophils and their impact on *L*. *sigmodontis* infection. Our study demonstrates that S100A8/A9 facilitates L3 migration. Mice deficient for S100A8/A9 had an increased inflammatory response within the BAL and thoracic cavity in response to the invading infective L3 larvae, which was associated with an increased neutrophil activation in these mice. Karadijan and colleagues previously demonstrated a pulmonary phase during *L*. *sigmodontis* L3 migration in susceptible WT BALB/c mice, which was characterized by inflamed endothelium and parenchyma as well as an increased number of neutrophils and an increased expression of S100A8/S100A9 [38]. These results are in accordance with our observations in C57BL/6 WT animals that were used as control for the S100A8/A9^-/-^ mice. *L*. *sigmodontis* infection in C57BL/6 WT controls further led to increased levels of S100A8/A9 in BAL as well as the thoracic cavity lavage in comparison to naïve controls.

S100A8/A9^-/-^ mice exhibited a decreased worm burden compared to WT controls following natural and subcutaneous *L*. *sigmodontis* infection, which was associated with an increased chemokine production in the BAL and thoracic cavity lavage. These results suggest that S100A8/A9 impairs the transient pulmonary inflammation that occurs during the migration of infective *L*. *sigmodontis* L3 larvae, which could facilitate larval migration. Comparable total numbers of neutrophils in the thoracic cavity and BAL of S100A8/A9^-/-^ mice upon *L*. *sigmodontis* infection is in contrast to previous studies demonstrating a S100A8 and S100A9-dependent recruitment of murine and human phagocytes to the site of inflammation. Such a neutrophil-recruiting function of S100A8 and S100A9 was demonstrated upon injection of S100A8 into the mouse foot pad, which led to an increase of neutrophils within the first 4–6 hours, followed by monocytes over 24 hours [61]. Furthermore, CXCL-1, CXCL-2, CXCL-5, and elastase were increased in the thoracic cavity and BAL of S100A8/A9^-/-^ mice compared to WT controls, which underlines that S100A8/A9 may regulate phagocyte recruitment and inflammation during filarial infection. Interestingly, another recent study showed, that calprotectin was able to exhibit protective effects in a murine experimental colitis model given by the intrarectal route, resulting in less macro- and microscopic damage, lower myeloperoxidase activity and decreased levels of pro-inflammatory cytokines such as TNF [59]. In contrast, S100A8/A9 seemed to be of less importance for the cellular immune response within the thoracic cavity and BAL as no changes in the analysed immune cell populations were observed between WT and S100A8/A9^-/-^ mice. Thus, the impact of S100A8/S100A9 may differ depending on the inflammatory milieu and location [2,3].

Analysis of the skin revealed that S100A8/A9^-/-^ mice had increased total cell counts of eosinophils, macrophages and neutrophils following intradermal L3 injection in comparison to WT controls. Previous studies from our labs reported that neutrophil recruitment during *L*. *sigmodontis* infection within the skin impairs L3 migration and leads to a reduced worm recovery, which is circumvented by subcutaneous or intravenous infection [40,41,48]. However, bypassing the epidermis and dermis during subcutaneous *L*. *sigmodontis* infection in S100A8/A9^-/-^ mice still resulted in a decreased worm burden in S100A8/A9^-/-^ mice. Following intravenous injections of the L3 larvae, which entirely bypasses protective immune responses in the skin as well as lymphatics [38], S100A8/A9^-/-^ mice had however an increased worm recovery in comparison to WT controls. Thus, intravenous L3 injection did not only annul the reduced worm burden observed in S100A8/A9^-/-^ mice following natural or subcutaneous infection, but further led to a significantly increased worm burden. Thus, we speculate that immune responses in the lower skin layers or the lymphatics mediate the protective effect in natural or subcutaneous infected S100A8/A9^-/-^ mice and/or the simultaneous entrance of the L3 larvae into the pulmonary capillaries upon intravenous L3 injection avoids the timely initiation of protective immune responses in S100A8/A9^-/-^ mice. With regard to changes in the lymphatics, analysis of the vascular permeability, which could facilitate L3 migration as well [58], did not reveal statistically significant differences in S100A8/A9^-/-^ and WT controls. Given that the worm recovery within the lung at 4dpi was already reduced in S100A8/A9^-/-^ mice further highlights that the elimination of L3 larvae occurs during the migration of the L3 larvae and before the entrance into the thoracic cavity.

Intranasal depletion of neutrophils in S100A8/A9^-/-^ animals during the migratory phase of *L*. *sigmodontis* increased the worm recovery from the thoracic cavity, indicating that neutrophils mediate protection in S100A8/A9^-/-^ animals, although the specific location of this neutrophil-dependent effect could not be determined as a systemic neutrophil depletion occurred.

The importance of neutrophils in the protective mechanism observed in S100A8/A9^-/-^ mice is further supported by the observed increased neutrophil activation, which arguably triggered an inflammatory milieu and supported the clearance of infective L3 larvae. This increased neutrophil activation in S100A8/A9^-/-^ mice was further associated with an increased apoptosis. According to the increased neutrophil activation, L3 larval motility was significantly reduced *in vitro* in the presence of S100A8/A9^-/-^ neutrophils compared to WT neutrophils in a DNA and NET formation-dependent manner, a mechanism that was previously described as neutrophil response to third stage larvae of several helminth species [41,62].

As S100A8/A9 is also expressed by monocytes, dendritic cells and mature macrophages [13,63,64], future studies should investigate their contribution during *L*. *sigmodontis* infection as well. In monocytes, 1% of the cytosolic proteins represent S100A8/A9 and its release has been associated with facilitated monocyte and neutrophil transmigration [65,66]. In accordance, absence of S100A9 reduced the responsiveness of monocytes to chemotaxis and IL-8-induced CD11b upregulation [53]. Thus, it is possible that in addition to neutrophils other S100A8/A9 expressing cells are contributing to the observed phenotype in S100A8/A9^-/-^ mice during *L*. *sigmodontis* infection. Another aspect that should be investigated in the future is the human relevance of S100A8/A9. S100A8/A9 has been previously investigated as marker for intestinal inflammation with controversial results so far [67,68]. A negative association between children infected with the intestinal helminth *Trichuris trichiura* and decreased intestinal inflammation as well as decreased concentration of S100A8/A9 compared to uninfected children has been reported [69]. In contrast, there was no evidence of an association between S100A8/A9 levels and *Schistosoma mansoni* [70] or *Ascaris lumbricoides* infection [71]. No impact of S100A8/A9 was also reported during urinary tract infection in S100A8/A9^-/-^ mice [72].

Collectively our data suggest that S100A8/A9 inhibits L3-induced inflammation by decreasing chemokine production and neutrophil activation. Given that the phenotype of the S100A8/A9^-/-^ mice with a reduced worm burden was abolished following neutrophil depletion and S100A8/A9 deficient neutrophils were shown to impair L3 motility more efficiently *in vitro*, it can be assumed that neutrophils are involved in the enhanced clearance of L3 larvae in S100A8/A9 deficient mice. Future studies will have to determine the exact role and importance of neutrophils in skin and lung in this process and whether other cells expressing S100A8/A9 are also contributing to the observed phenotype.

## Supporting information

S1 Fig Gating strategy to identify cell populations of interest. Shown is a representative gating strategy from thoracic cavity and bronchoalveolar cells of a wildtype mouse infected for 12 days with *L*. *sigmodontis*. All cells were gated and analysed by FSC/SSC and duplicates were removed by FSC-W/SSC-A. Singlets (P2 gate) were analysed for their expression of CD4, CD8, Ly6G, CD11b, SiglecF and F4/80 to differentiate CD4+ (CD4+ CD8-), CD8+ (CD8+ CD4-) T cells, neutrophils (Ly6G+, CD11b+), eosinophils (SiglecF+ F4/80 low) and macrophages (SiglecF low/int F4/80+). SiglecF- F4/80- cells (P3 gate) were analyzed for their expression of Ly6G to identify neutrophils. Bronchoalveolar macrophages were identified by their expression of SiglecF+/F4/80+. Gating was performed using the fluorescence minus one (FMO) approach.
(TIFF)

S2 Fig Systemic neutrophil depletion by intranasal anti-Ly6G antibody treatment. Frequency of neutrophils within the bronchoalveolar lavage (BAL), thoracic cavity lavage, blood, spleen and skin in naïve WT mice treated either with an isotype control or with an anti-Ly6G depleting antibody. Results are shown as median (**A-E**). Statistical significance was analyzed by two-tailed non-parametric Mann-Whitney-U-test (**A-D**) and by Kruskal-Wallis followed by Dunn’s multiple comparison test (**E**). One experiment with 3 mice per group.
(TIFF)

S3 Fig Spleen cell responses 12 days after *L. sigmodontis* infection. Total number of spleen cells in WT and S100A8/A9^-/-^ mice 12 days after subcutaneous *L*. *sigmodontis* infection. Total number of splenocytes (**A**), CD4+ T cells (CD4+ T), CD8+ T cells (CD8+ T), neutrophils (NØ), macrophages (Macs), and eosinophils (Eos, **B**). Concentrations of IFN-γ (**C**) and TNF (**D**) after *in vitro* spleen cell culture without stimulation (US) or after stimulation with ConA or *L*. *sigmodontis* extract (LsAg) of WT and S100A8/A9^-/-^ mice 12 days after subcutaneous *L*. *sigmodontis* infection. Results are shown as median. Statistical significance was analyzed by two-tailed non-parametric Mann-Whitney-U-test. *p<0.05, **p<0.01, ***p<0.001. Data are pooled from two independent experiments (**A-B**) with 8 mice per group. Data shown in **C** and **D** are pooled from two independent experiments with 4 mice per group.
(TIFF)

S4 Fig Reduced worm burden, but increased neutrophil mediator release following natural *L. sigmodontis* infection in S100A8/A9-/- mice. Worm burden following natural *L*. *sigmodontis* infection in WT and S100A8/A9^-/-^ mice at 12 days after infection (**A**). Number of thoracic cavity cells (**B**) and total cell count of the thoracic cavity lavage 12 days after *L*. *sigmodontis* infection in WT and S100A8/A9^-/-^ mice showing CD4+ T cells (CD4^+^ T), CD8+ T cells (CD8^+^ T), neutrophils (NØ), macrophages (Macs) and eosinophils (Eos) (**C**). Concentrations of CXCL-1 (**D**), CXCL-2 (**E**), CXCL-5 (**F**), and elastase (**G**) in the thoracic cavity lavage 12 days after *L*. *sigmodontis* infection in WT and S100A8/A9^-/-^ mice. Results are shown as median (**A**-**G**). Statistical significance was analyzed by two-tailed non-parametric Mann-Whitney-U-test. *p<0.05, **p<0.01. One experiment with 5 mice per group.
(TIFF)

S5 Fig Impact of S100A8/A9 on worm recovery and cellular responses in the thoracic cavity 29 days after *L. sigmodontis* infection. Worm burden in WT and S100A8/A9^-/-^ mice (**A**) and total number of thoracic cavity cells (**B**) and total cell counts of neutrophils (NØ), macrophages (Macs) and eosinophils (Eos) in the thoracic cavity lavage (**C**) 29 days after natural *L*. *sigmodontis* infection. Results are shown as median (**A-C**). Statistical significance was analyzed by two-tailed non-parametric Mann-Whitney-U-test (**A-C**). *p<0.05, **p<0.01. Data shown is of one experiment with 9–10 mice per group.
(TIFF)

S6 Fig Intradermal injection of infective *L. sigmodontis* L3 larvae leads to increased cellular immune responses within the skin of S100A8/A9^-/-^ mice. Total number of skin cells (**A**), eosinophils (**B**), neutrophils (**C**), and macrophages (**D**) and their respective MHCII expression (**E**, **F**, **G**) in WT and S100A8/A9^-/-^ mice 3h after intradermal *L*. *sigmodontis* L3 or PBS injection. Results are shown as median. Statistical significance of not normally distributed data (**A**-**G**) was analyzed by Kruskal-Wallis followed by Dunn’s multiple comparison test. p<0.05, **p<0.01. Representative data from one out of two independent experiments with 5 mice per group (A-G).
(TIFF)

S7 Fig Migrating L3 larvae cause transient pulmonary inflammation. C57BL/6 WT and S100A8/A9^-/-^ mice were naturally infected with *L*. *sigmodontis*. Representative pictures of a WT mouse lung 12 days after natural *L*. *sigmodontis* infection (**A**) and an uninfected WT mouse (**B**). Pathology of lung blood vessels (bv) and mucus production in 12-day *L*. *sigmodontis*-infected mice (**C**) (left panel) and naïve mice (**D**) (right panel; br = bronchus; pvs = perivascular space). Number of congested blood vessels (**E**) and bronchi with goblet cells (**F**) from naïve and 12-day *L*. *sigmodontis*-infected WT and S100A8/A9^-/-^ mice. Spearman correlation of congested blood vessels and worm counts (**G**) as well as bronchi with goblet cells and worm burden (**H**). Total number of cells in PVS in intact and congested blood vessels of WT and S100A8/A9^-/-^ mice 12 days after *L*. *sigmodontis* infection, as well as naïve controls (**I**). Influx of neutrophils (**J, K**) and eosinophils (**L, M**) in the perivascular space (PVS) of congested (**J, L**) and intact blood vessels (**K, M**) of 12-day *L*. *sigmodontis*-infected WT mice. Results are shown as median (**E, F, I**). Data are pooled from 2 independent experiments (**G, H**) with 3–8 mice per group. Remaining figures are representatives of two experiments with at least 2–5 mice per group.
(TIFF)
