# Supplementary figures and images for: S100A8/S100A9 deficiency increases neutrophil activation and protective immune responses against invading infective L3 larvae of the filarial nematode Litomosoides sigmodontis
Source: PLoS Negl Trop Dis. 2020 Feb 27;14(2):e0008119. doi: 10.1371/journal.pntd.0008119 (PMC7064255; doi:10.1371/journal.pntd.0008119)

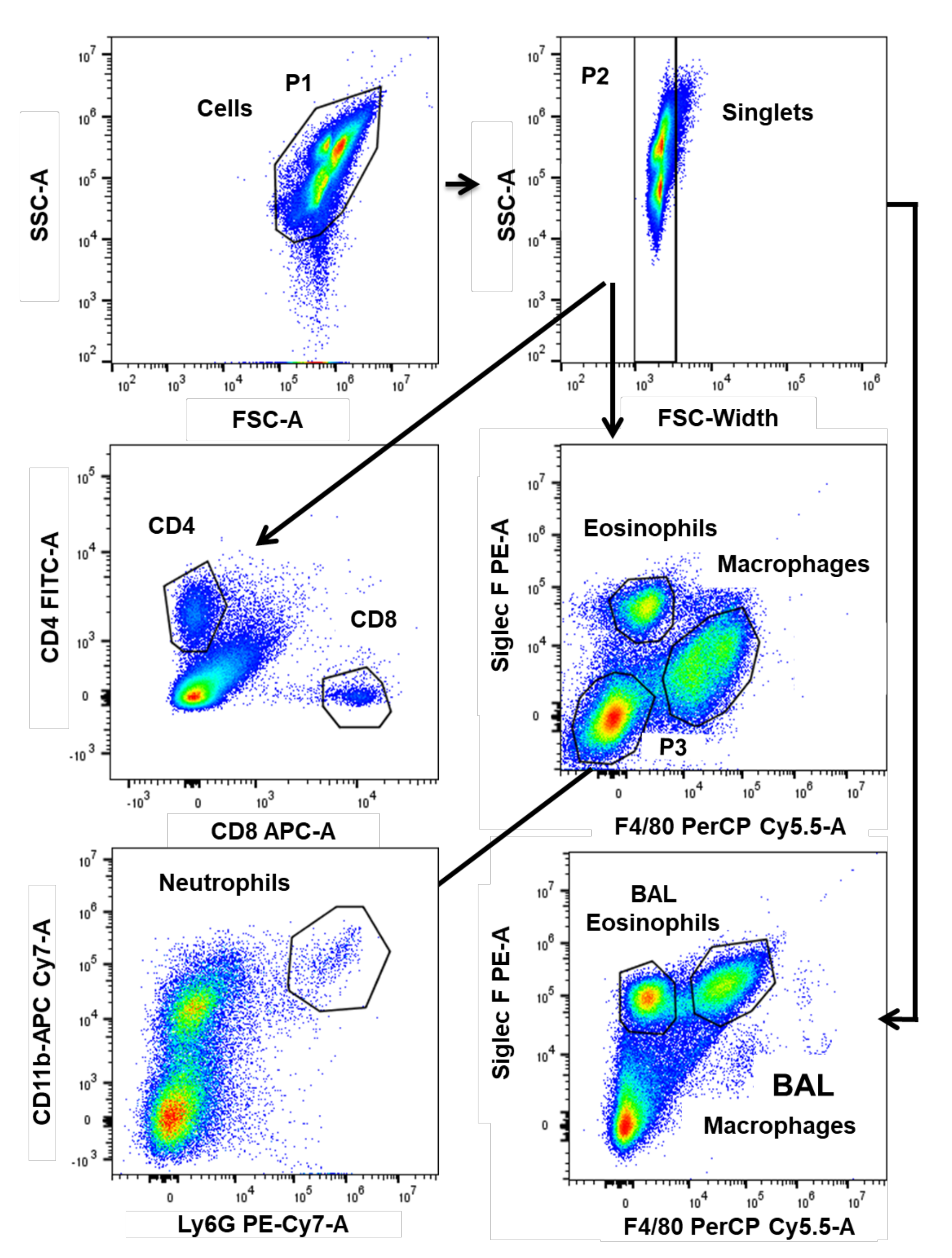

Supplement: S1 Fig — Shown is a representative gating strategy from thoracic cavity and bronchoalveolar cells of a wildtype mouse infected for 12 days with L. sigmodontis. All cells were gated and analysed by FSC/SSC and duplicates were removed by FSC-W/SSC-A. Singlets (P2 gate) were analysed for their expression of CD4, CD8, Ly6G, CD11b, SiglecF and F4/80 to differentiate CD4+ (CD4+ CD8-), CD8+ (CD8+ CD4-) T cells, neutrophils (Ly6G+, CD11b+), eosinophils (SiglecF+ F4/80 low) and macrophages (SiglecF low/int F4/80+). SiglecF- F4/80- cells (P3 gate) were analyzed for their expression of Ly6G to identify neutrophils. Bronchoalveolar macrophages were identified by their expression of SiglecF+/F4/80+. Gating was performed using the fluorescence minus one (FMO) approach. (TIFF) [file pntd.0008119.s001.tiff]

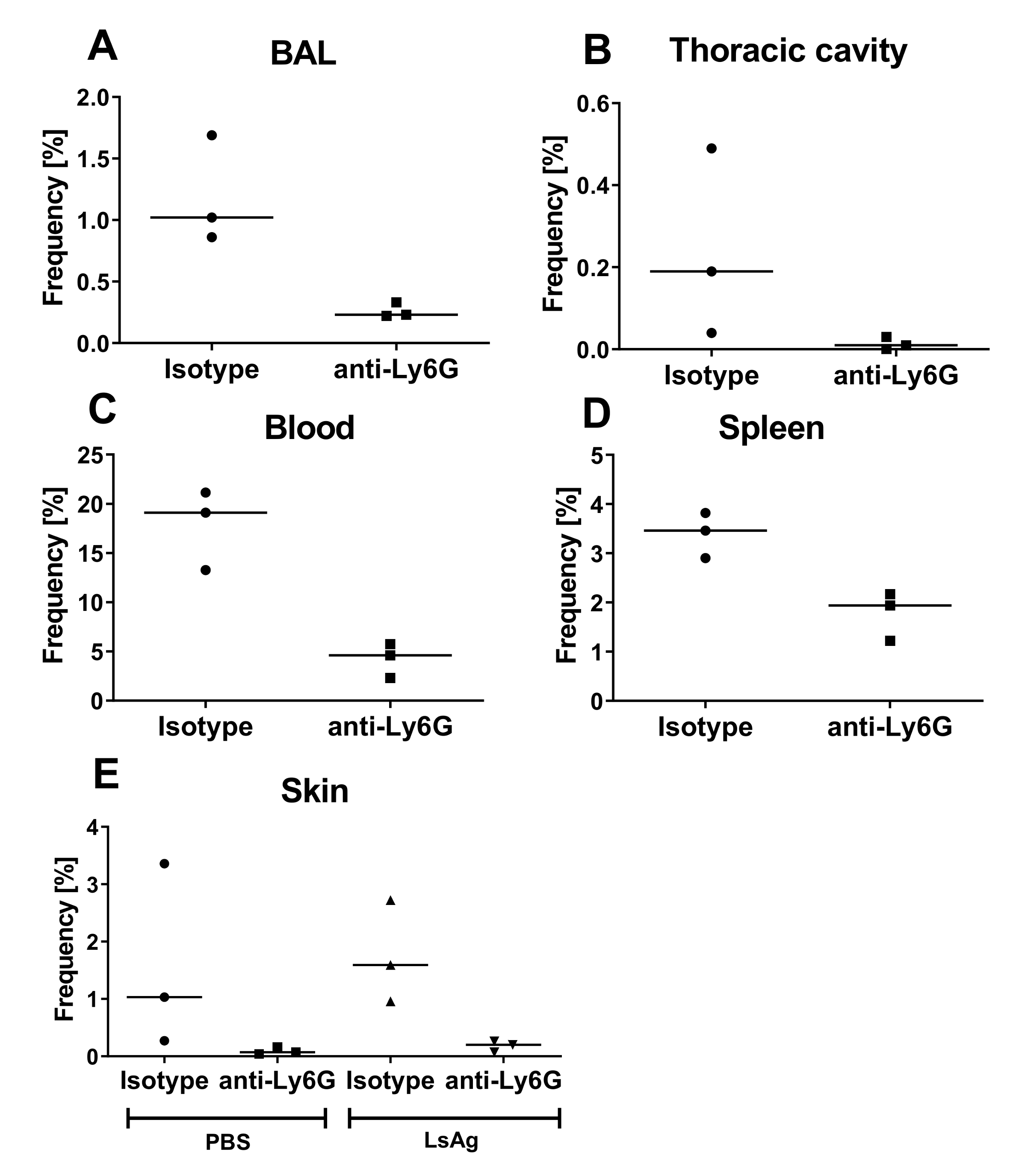

Supplement: S2 Fig — Frequency of neutrophils within the bronchoalveolar lavage (BAL), thoracic cavity lavage, blood, spleen and skin in naïve WT mice treated either with an isotype control or with an anti-Ly6G depleting antibody. Results are shown as median (A-E). Statistical significance was analyzed by two-tailed non-parametric Mann-Whitney-U-test (A-D) and by Kruskal-Wallis followed by Dunn’s multiple comparison test (E). One experiment with 3 mice per group. (TIFF) [file pntd.0008119.s002.tiff]

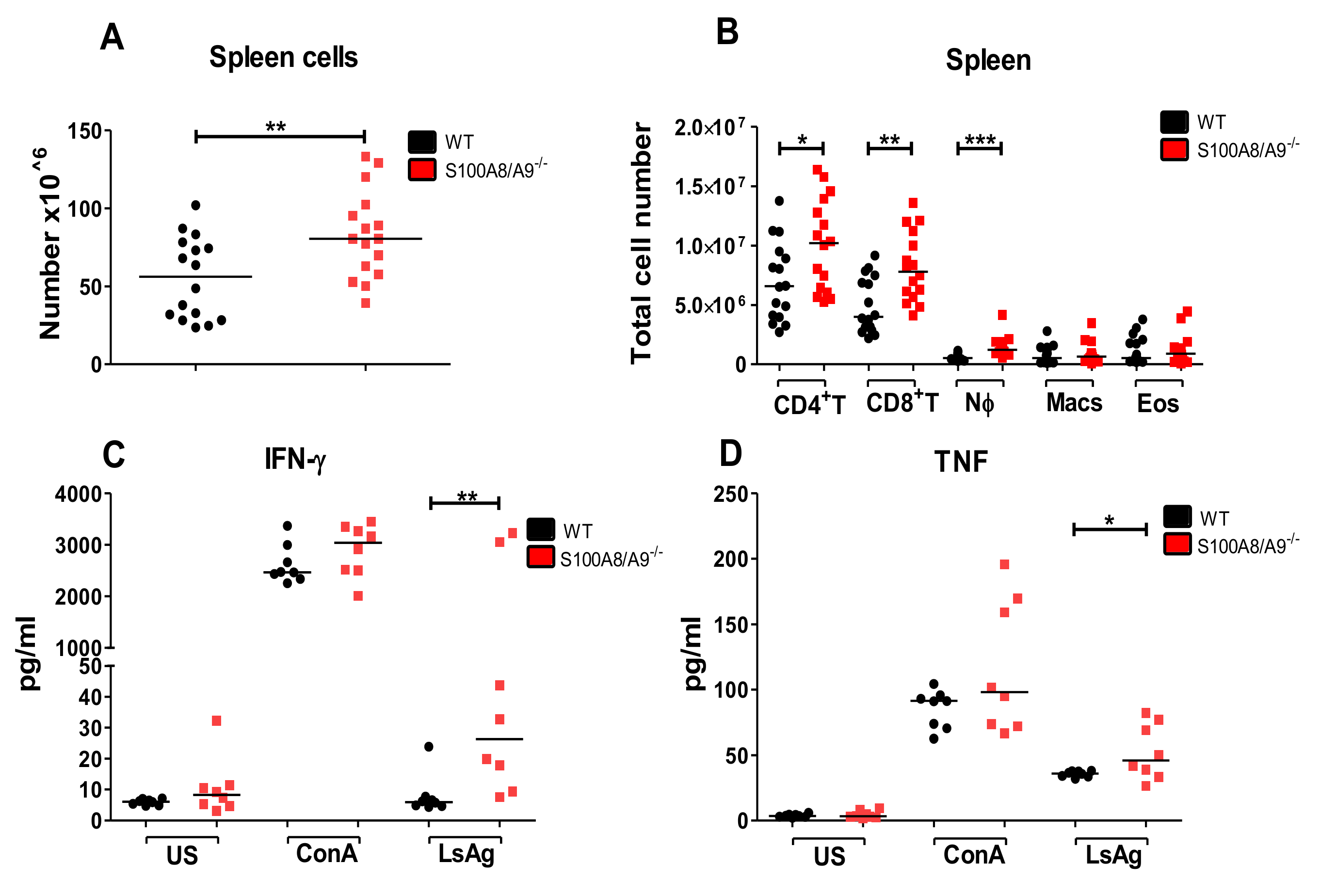

Supplement: S3 Fig — Total number of spleen cells in WT and S100A8/A9-/- mice 12 days after subcutaneous L. sigmodontis infection. Total number of splenocytes (A), CD4+ T cells (CD4+ T), CD8+ T cells (CD8+ T), neutrophils (NØ), macrophages (Macs), and eosinophils (Eos, B). Concentrations of IFN-γ (C) and TNF (D) after in vitro spleen cell culture without stimulation (US) or after stimulation with ConA or L. sigmodontis extract (LsAg) of WT and S100A8/A9-/- mice 12 days after subcutaneous L. sigmodontis infection. Results are shown as median. Statistical significance was analyzed by two-tailed non-parametric Mann-Whitney-U-test. *p<0.05, **p<0.01, ***p<0.001. Data are pooled from two independent experiments (A-B) with 8 mice per group. Data shown in C and D are pooled from two independent experiments with 4 mice per group. (TIFF) [file pntd.0008119.s003.tiff]

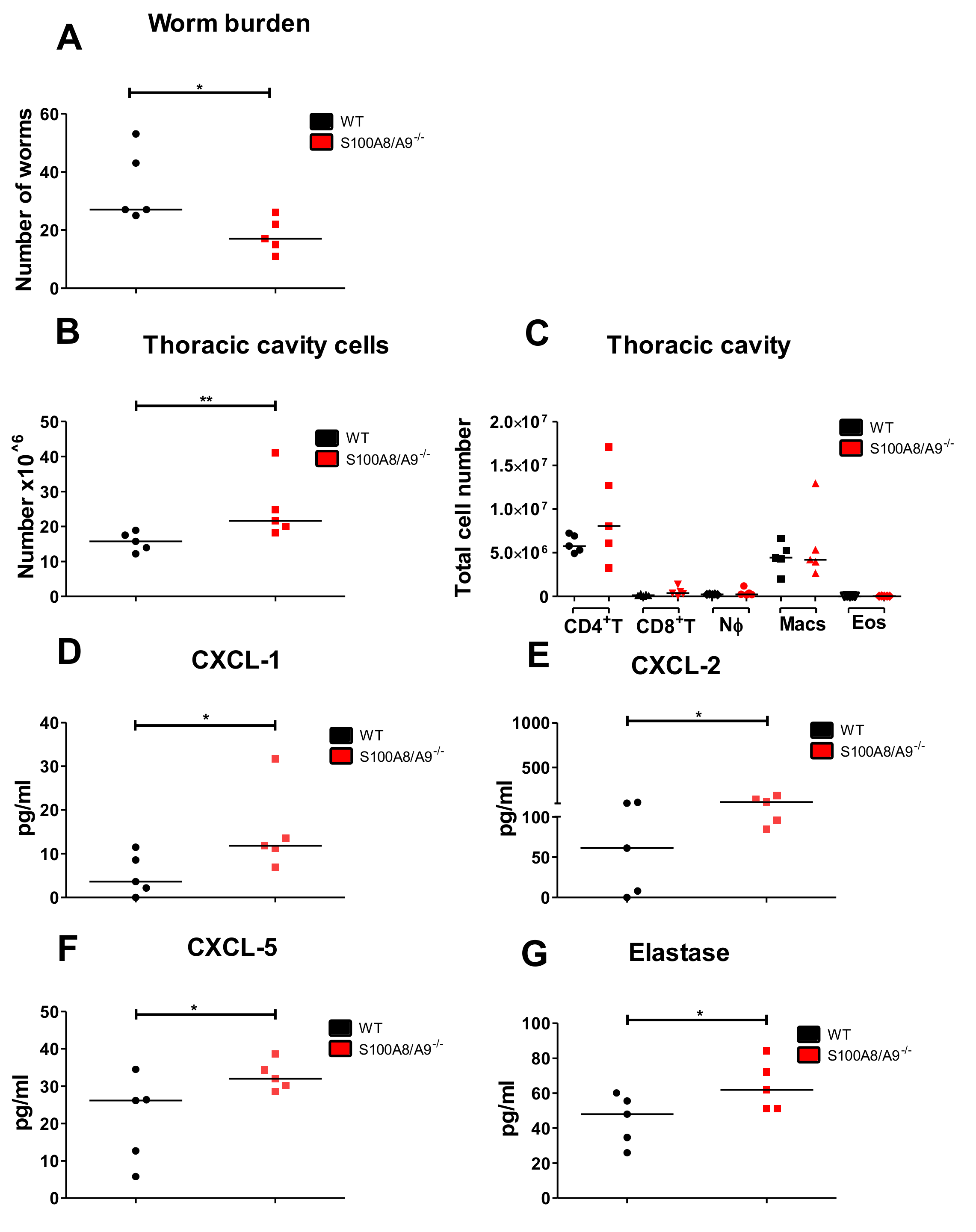

Supplement: S4 Fig — Worm burden following natural L. sigmodontis infection in WT and S100A8/A9-/- mice at 12 days after infection (A). Number of thoracic cavity cells (B) and total cell count of the thoracic cavity lavage 12 days after L. sigmodontis infection in WT and S100A8/A9-/- mice showing CD4+ T cells (CD4+ T), CD8+ T cells (CD8+ T), neutrophils (NØ), macrophages (Macs) and eosinophils (Eos) (C). Concentrations of CXCL-1 (D), CXCL-2 (E), CXCL-5 (F), and elastase (G) in the thoracic cavity lavage 12 days after L. sigmodontis infection in WT and S100A8/A9-/- mice. Results are shown as median (A-G). Statistical significance was analyzed by two-tailed non-parametric Mann-Whitney-U-test. *p<0.05, **p<0.01. One experiment with 5 mice per group. (TIFF) [file pntd.0008119.s004.tiff]

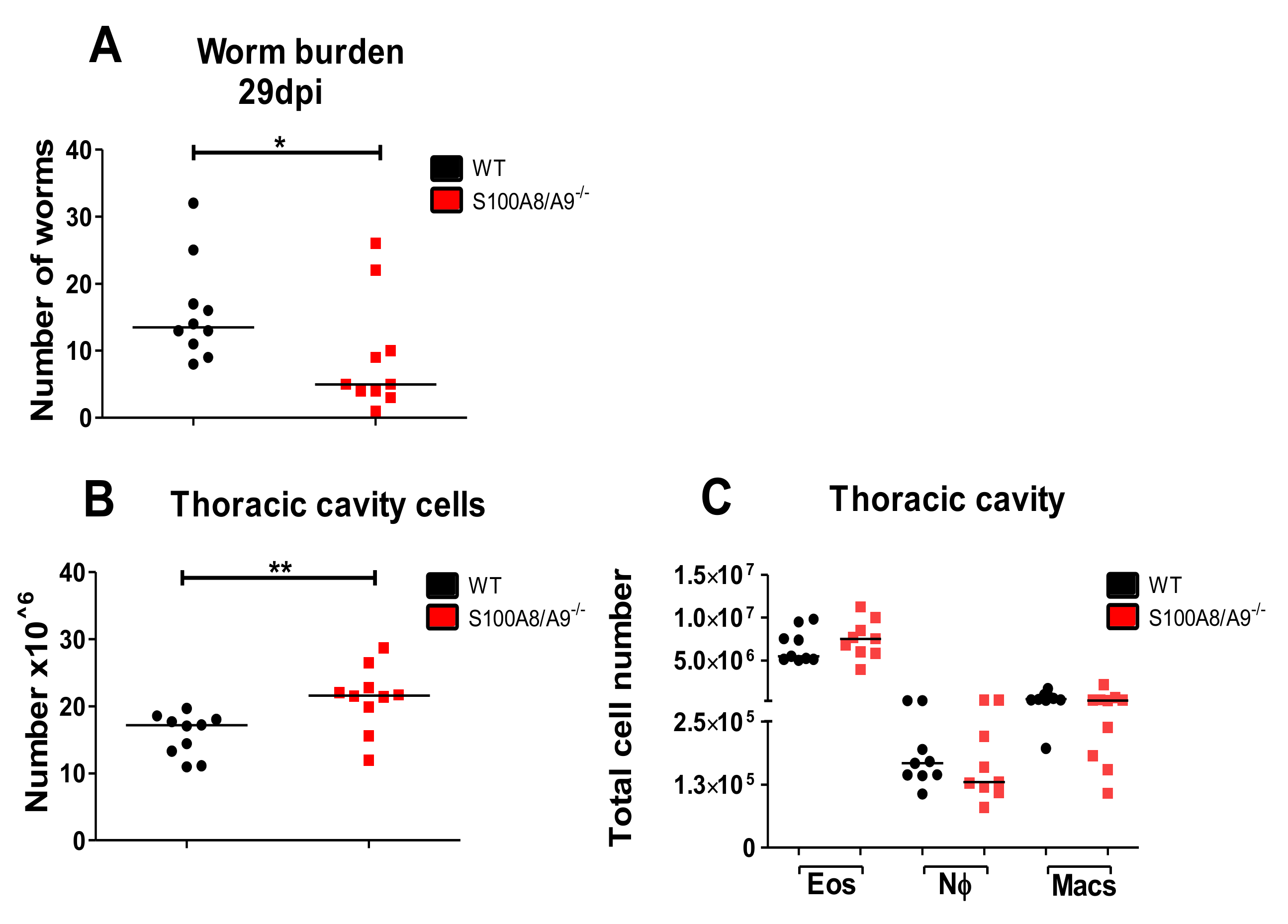

Supplement: S5 Fig — Worm burden in WT and S100A8/A9-/- mice (A) and total number of thoracic cavity cells (B) and total cell counts of neutrophils (NØ), macrophages (Macs) and eosinophils (Eos) in the thoracic cavity lavage (C) 29 days after natural L. sigmodontis infection. Results are shown as median (A-C). Statistical significance was analyzed by two-tailed non-parametric Mann-Whitney-U-test (A-C). *p<0.05, **p<0.01. Data shown is of one experiment with 9–10 mice per group. (TIFF) [file pntd.0008119.s005.tiff]

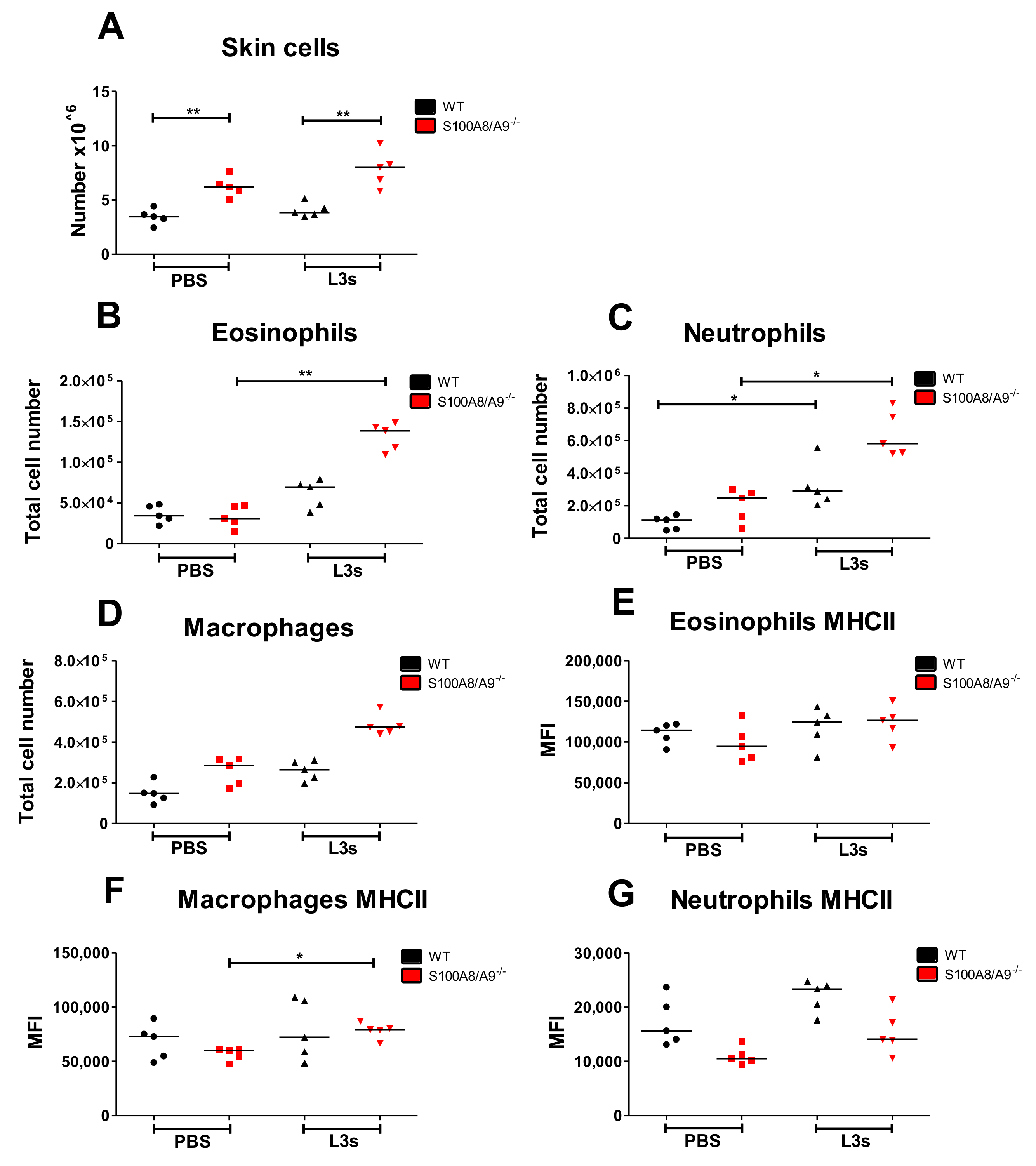

Supplement: S6 Fig — Total number of skin cells (A), eosinophils (B), neutrophils (C), and macrophages (D) and their respective MHCII expression (E, F, G) in WT and S100A8/A9-/- mice 3h after intradermal L. sigmodontis L3 or PBS injection. Results are shown as median. Statistical significance of not normally distributed data (A-G) was analyzed by Kruskal-Wallis followed by Dunn’s multiple comparison test. p<0.05, **p<0.01. Representative data from one out of two independent experiments with 5 mice per group (A-G). (TIFF) [file pntd.0008119.s006.tiff]

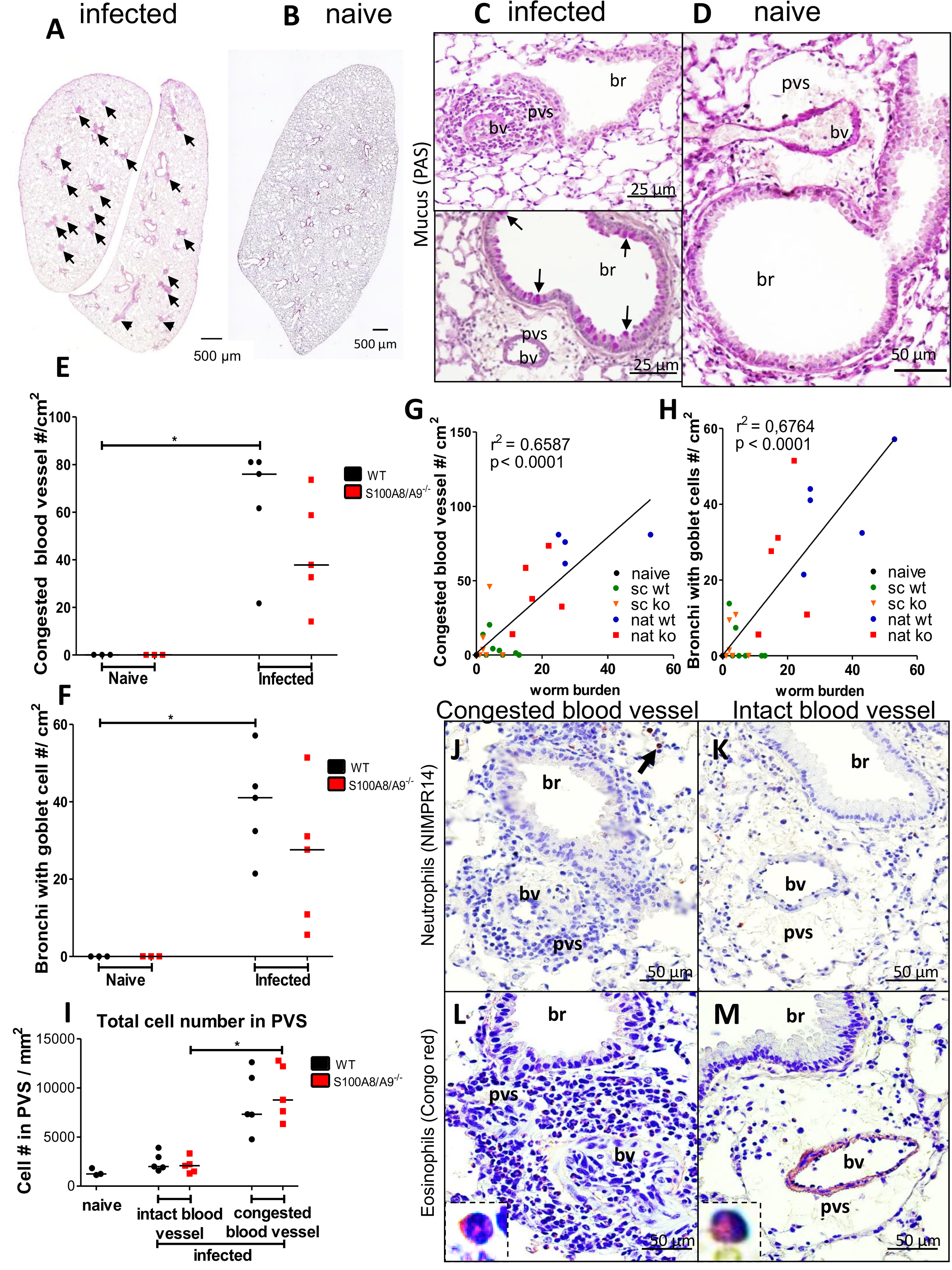

Supplement: S7 Fig — C57BL/6 WT and S100A8/A9-/- mice were naturally infected with L. sigmodontis. Representative pictures of a WT mouse lung 12 days after natural L. sigmodontis infection (A) and an uninfected WT mouse (B). Pathology of lung blood vessels (bv) and mucus production in 12-day L. sigmodontis-infected mice (C) (left panel) and naïve mice (D) (right panel; br = bronchus; pvs = perivascular space). Number of congested blood vessels (E) and bronchi with goblet cells (F) from naïve and 12-day L. sigmodontis-infected WT and S100A8/A9-/- mice. Spearman correlation of congested blood vessels and worm counts (G) as well as bronchi with goblet cells and worm burden (H). Total number of cells in PVS in intact and congested blood vessels of WT and S100A8/A9-/- mice 12 days after L. sigmodontis infection, as well as naïve controls (I). Influx of neutrophils (J, K) and eosinophils (L, M) in the perivascular space (PVS) of congested (J, L) and intact blood vessels (K, M) of 12-day L. sigmodontis-infected WT mice. Results are shown as median (E, F, I). Data are pooled from 2 independent experiments (G, H) with 3–8 mice per group. Remaining figures are representatives of two experiments with at least 2–5 mice per group. (TIFF) [file pntd.0008119.s007.tiff]
